# Comparative analysis of plan quality between two SRS systems for stereotactic irradiation in treating Vestibular Schwannoma

**DOI:** 10.1002/acm2.70354

**Published:** 2025-11-18

**Authors:** Toshihiro Suzuki, Masahide Saito, Ryutaro Nomura, Hikaru Nemoto, Ryuma Sawada, Tran Van Ton, Zhe Chen, Zennosuke Mochizuki, Naoki Sano, Hiroshi Onishi, Hiroshi Takahashi

**Affiliations:** ^1^ CyberKnife Center Kasugai General Rehabilitation Hospital Yamanashi Japan; ^2^ Department of Radiology University of Yamanashi Yamanashi Japan; ^3^ Department of Neurosurgery Kamiyacho Neurosurgical Clinic Tokyo Japan; ^4^ Department of Radiotherapy, Oncology Center Military Hospital 103 Hanoi Vietnam; ^5^ Department of Neurosurgery Kasugai General Rehabilitation Hospital Yamanashi Japan

**Keywords:** CyberKnife, vestibular schwannoma, ZAP‐X

## Abstract

**Purpose:**

The purpose of this study was to compare plan quality for vestibular schwannoma (VS) between CyberKnife (CK) and ZAP‐X.

**Methods:**

Thirty VS cases treated with CK were re‐planned on CK and newly planned on ZAP‐X. Both plans ensured 98% target coverage. The 30 patients were divided into two groups: 15 patients with Koos grade I and II (grade I/II group) and 15 patients with Koos grade III and IV (grade III/IV group). For patients in the grade I/II group, 12 Gy in 1 fraction was prescribed. For those in the grade III/IV group, they received the total dose of 21 Gy in three fractions. The evaluated parameters included the Gradient Index (GI), Paddick Conformity Index (CI), cochlea and brainstem doses, monitor units (MU), and beam‐on time (BT).

**Results:**

In the results, ZAP‐X achieved lower mean GI in the grade I/II group while CK did better in the grade III/IV group. The mean CI was higher for ZAP‐X in both groups, with a significant difference. The mean cochlear and brainstem doses were lower with ZAP‐X in the grade I/II group, with a significant difference. In contrast, no significant difference was observed in the grade III/IV group. In both groups, the mean MU was lower for CK, and the mean BT was shorter for CK.

**Conclusion:**

In radiation therapy for VS, ZAP‐X can provide a treatment plan comparable to that of CK. Notably, for smaller targets, ZAP‐X has the potential to achieve superior treatment planning compared to CK.

## 1 INTRODUCTION

Vestibular schwannoma (VS) is benign tumor arising from Schwann cells of the vestibulocochlear nerve, accounting for approximately 6%–8% of all intracranial tumors, with an annual incidence of around 3 to 5 cases per 100 000 individuals.^1^, ^2^ VS presents either as a sporadic unilateral tumors or as bilateral hereditary tumors associated with neurofibromatosis type 2 (NF2)^3^ Management options include observation, microsurgery, or radiosurgery, with treatment selection depending on tumor size and proximity to critical structures.^4^, ^5^ The Koos grading scale is commonly used to guide treatment decisions.^6^ Stereotactic radiosurgery (SRS) is generally recommended for Koos grade I and II, while surgery is often indicated for grade IV.^7^ However, several studies have also demonstrated favorable outcomes with SRS in patients with grade III and IV.^8^, ^9^, ^10^ The choice between SRS or stereotactic radiotherapy (SRT) for radiation therapy is still being debated.^11^, ^12^ Multiple radiation therapy modalities have been applied to VS, including Gamma Knife (GK; Elekta Instruments AB, Sweden), CyberKnife (CK; Accuray, Sunnyvale, CA), conventional LINAC‐based methods such as volumetric modulated arc therapy (VMAT) and intensity‐modulated radiation therapy (IMRT), Helical TomoTherapy (HT; Accuray, Sunnyvale, CA), and ZAP‐X (ZAP‐X; Zap Surgical Systems Inc., San Carlos, CA). Studies utilizing these techniques have been reported.^13^, ^14^, ^15^, ^16^, ^17^, ^18^ Among these techniques, CK is a SRS and SRT platform that delivers multiple isocentric or non‐isocentric photon beams to the target through a 6 MV linear accelerator mounted on a robotic arm. Additionally, CK can acquire 2D images every 5–150 s using 2‐kV x‐ray devices (target locating system [TLS]), enabling high‐precision image‐guided radiotherapy.^19^ ZAP‐X is a fully self‐shielded device recently developed for the treatment of intracranial and cervical spine lesions and uses a 3 MV linear accelerator.^20^ The 3 MV x‐rays are delivered to the target at a dose rate of 1500 MU/min, with a short source‐to‐axis distance (SAD) of 450 mm. Eight collimator sizes are available, ranging from 4 to 25 mm. During irradiation, the actual transmitted dose can be monitored in real‐time using an MV imager positioned diagonally within the linear accelerator. Furthermore, precise image guidance is facilitated by the kV imaging system mounted on the gantry. Both CK and ZAP‐X utilize compact LINAC designs, offering non‐coplanar radiation delivery and enabling the achievement of optimized treatment plans desirable for intracranial SRS.^21^

To the best of our knowledge, no studies have compared CK and ZAP‐X, including their effects on target volume. The purpose of this study is to compare the dosimetric and delivery parameters in the treatment planning of VS cases classified by the Koos grading scale using CK and ZAP‐X.

## 2 MATERALS AND METHODS

### 2.1 Patient selection

This retrospective study was approved by the institutional review board of our hospital (approval number: 2024‐2). The study included 30 patients who underwent radiotherapy for VS using CK between October 2015 and October 2024. Patients with NF2 or a history of prior surgery and/or radiation therapy were excluded. Informed consent was obtained using an opt‐out methodology publicly disclosed on the hospital's website.

### 2.2 Planning method

All computed tomography (CT) images were acquired using the Optima CT660 scanner (GE Medical Systems, Milwaukee, WI) with the following imaging parameters: 120 kV, 400 mA, 1.25‐mm slice thickness, 300‐mm field of view, and a resolution of 512 × 512 pixels. All structures, including the target volume, cochlea, brainstem, and skin (3 mm thick) were contoured based on planning CT images and magnetic resonance imaging. The target volume was delineated by a neurosurgeon with extensive experience in CK treatment. In this study, the planning target volume was identical to the gross tumor volume, as no margin was applied. CT images and contoured structures of selected patients were transferred to the respective treatment planning systems (TPS) via DICOM‐RT. Treatment plans were generated by a neurosurgeon and a medical physicist using their respective TPS. All plans were designed to achieve >98% target coverage and to meet organs at risk (OAR) dose constraints proposed by Timmerman et al.^22^ Cochlear dose constraints were applied in all cases, irrespective of hearing status. Thirty patients were divided into two groups: 15 patients with Koos grade I and II (grade I/II group) and 15 patients with Koos grade III and IV (grade III/IV group). The prescription dose and number of fractions were 12 Gy/1 fraction for the grade I/II group and 21 Gy/3 fractions for the grade III/IV group. CK plans were replanned using CK MultiPlan TPS version 3.2.0 (Accuray). The CK G4 system utilizes Fixed or IRIS collimators selected according to target volume and geometry. To refine dose distribution, two to three dose‐shaping shells were placed outside the target volume, with planning performed by sequential optimization. Prescription doses were calculated using inverse planning with the ray‐tracing algorithm. ZAP‐X plans were planned using ZAP‐X TPS version 1.8.58.12369 (Zap Surgical Systems). A non‐coplanar beam arrangement was automatically optimized by the TPS. Collimator sizes were selected based on target volume and shape, with dynamic collimation applied to enhance dose conformity. This system enables real‐time, seamless adjustments of collimator size to optimize dose conformity and minimize normal tissue exposure. Dose distribution was optimized using beam weighting across varying gantry angles, employing a combination of forward and inverse planning techniques. Prescription doses were calculated using the ray‐tracing algorithm, ensuring efficient and accurate dose computation. All treatment plans were independently reviewed and confirmed by two radiation oncologists prior to finalization.

### 2.3 Comparison parameters

Dosimetric parameters, including the dose (*D*_98%_ and *D*_2%_), Gradient Index (GI), and Paddick Conformity Index (CI), were compared in each group. OAR doses were compared in each group using *D*_max_ for cochlea, *D*_1cc_ for the brainstem, *D*_10cc_ for the skin, and V7 Gy and V12 Gy for normal brain tissue.

Delivery parameters, including monitor units (MU) and beam‐on time (BT), were also compared in each group.

GI was defined as $GI=PIV_{50\%}/\;PIV$ where PIV_50%_ represents the volume covered by 50% prescription dose and PIV represents the volume covered by the prescription dose. GI is used to reflect the decrease in dose outside the target volume.

CI was defined as $CI={\left(TV_{PIV}\right)}^{2}/TV\times PIV$ where TV*_PIV_* was the volume of the target covered by the prescription isodose line; TV was the target volume. The maximum CI value is 1.0; the closer it is to 1, the better the target fit. If the target is fully covered by the prescription dose line, the CI equals 1.0. For each parameter, the mean values and standard deviations (SD) were calculated for each group. All dosimetry parameters were calculated using MIM Maestro ver. 7.2.9 (MIM Software, Inc., Cleveland, OH) and the delivery parameters were obtained from each TPS. Additionally, due to the significant difference in photon energy between CK and ZAP‐X, the integral dose (ID) to the normal brain tissue was evaluated. ID was defined as $\mathrm{ID}\;\left[\mathrm{Gy}\cdot L\right]=D_{\mathrm{mean}}\;\times V$ where *D*_mean_ was the mean dose of the normal brain and V was its volume.^23^, ^24^

### 2.4 Statistical analysis

The Wilcoxon signed‐rank test was used to compare values between CK and ZAP‐X for each parameter. All data analyses were performed using RStudio (RStudio: Integrated Development for R. RStudio, PBC, Boston, MA). A *p*‐value of less than 0.05 was considered statistically significant.

## 3 RESULTS

Table 1 shows the characteristics of the 15 patients in each group. In the grade I/II group, the mean target volume (range) was 0.49 cc (0.11–0.99) with six Koos grade I cases and nine grade II cases. In the grade III/IV group, the mean target volume (range) was 4.61 cc (2.04–8.06), including seven grade III cases and eight grade IV cases.

**TABLE 1:** Characteristics of the 15 patients in the grade I/ II and III/IV group, respectively.

	Grade I/ II group	Grade III/IV group
Patients (n)	15	15
Sex (M:F)	7:8	8:7
Age (years) Median [range]	65 [50–85]	67 [24–86]
Target side (right:Left)	6:9	8:7
Koos grade	–	–
1:2	6:9	–
3:4	–	7:8
Mean target volume (cc) [range]	0.49 [0.11–0.99]	4.61 [2.04–8.06]

**FIGURE 1:**
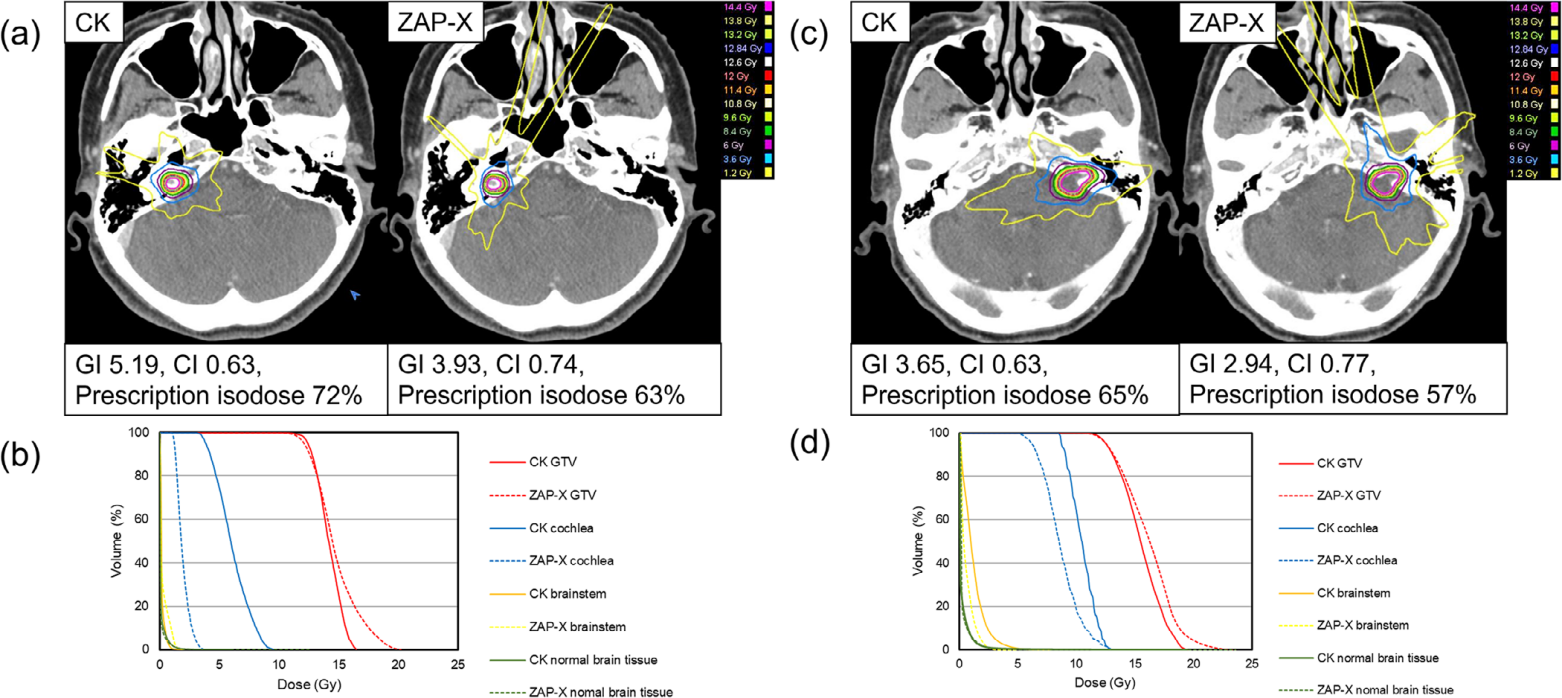
Dose distribution and dose‐volume histogram (DVH) for representative cases in the grade I/II group: (a) dose distribution for Koos grade I, (b) DVH for Koos grade I, (c) dose distribution for Koos grade II, and (d) DVH for Koos grade II. The Koos grade I case corresponds to Patient 2 (Target volume: 0.22 cc, prescription dose: 12 Gy), while the Koos grade II case corresponds to Patient 6 (Target volume: 0.97 cc, prescription dose: 12 Gy).

**FIGURE 2:**
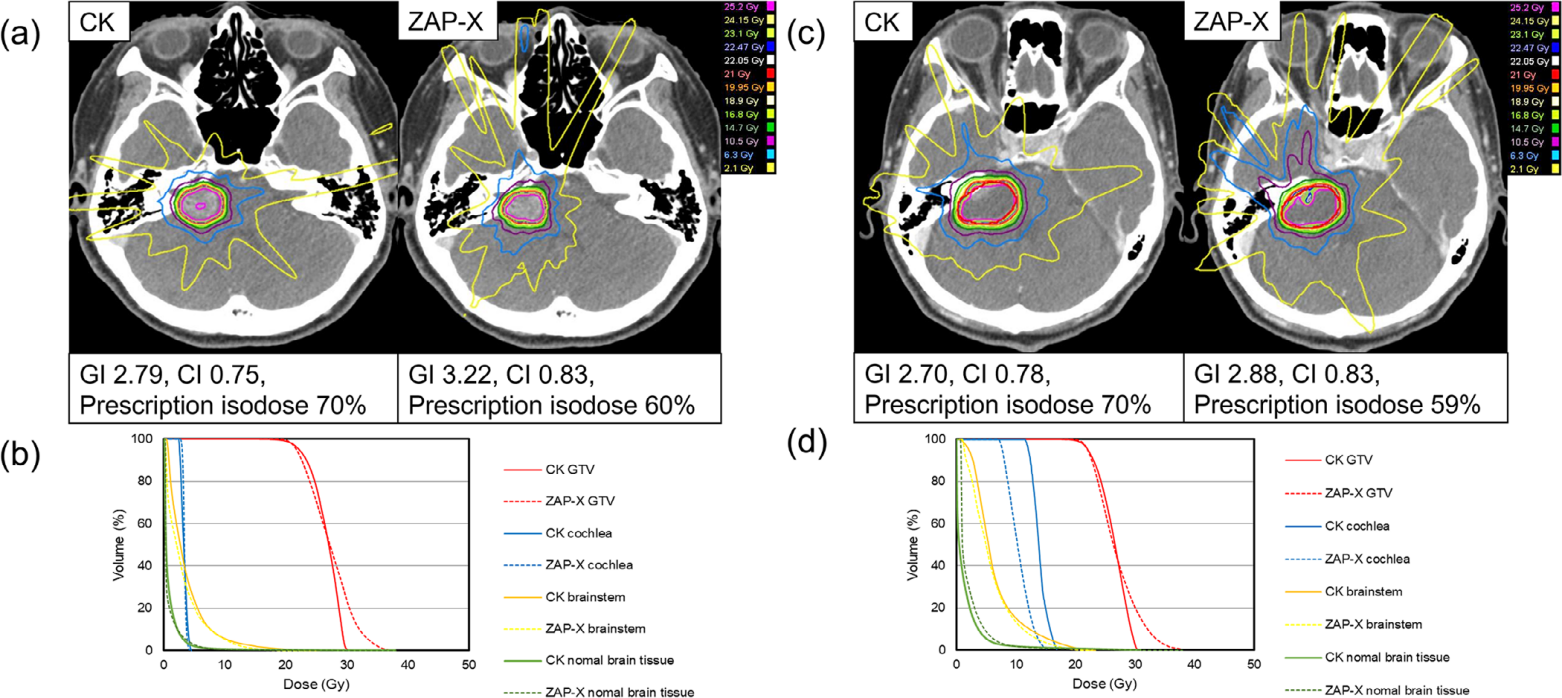
Dose distribution and DVH for representative cases in the grade III/IV group: (a) dose distribution for Koos grade III, (b) DVH for Koos grade III, (c) dose distribution for Koos grade IV, and (d) DVH for Koos grade IV. The Koos grade III case corresponds to Patient 18 (Target volume: 2.62 cc, prescription dose: 21 Gy), while the Koos grade IV case corresponds to Patient 28 (Target volume: 8.06 cc, prescription dose: 21 Gy).

Table 2 shows the dosimetric and delivery parameters (mean ± SD). In the grade I/II group, the prescription isodose was significantly higher with CK than with ZAP‐X (70% vs. 57%, *p* < 0.05). ZAP‐X achieved a lower GI and higher CI than CK (*p* < 0.05). Target D98% was below 12 Gy with ZAP‐X, while target D2% was higher compared with CK (*p* < 0.05). Cochlear, brainstem, skin doses, and V7 Gy / V12 Gy of normal brain were significantly lower with ZAP‐X (*p* < 0.05). Furthermore, no significant difference was observed in the ID of the normal brain (*p* = 1.00). In contrast, MU was lower with CK (*p* < 0.05). BT tended to be shorter with CK, but the difference was not significant (*p* = 0.118). In the grade III/IV group, the prescription isodose was again higher with CK (69% vs. 57%, *p* < 0.05). GI was significantly lower with CK, while CI was higher with ZAP‐X (*p* < 0.05). Both modalities delivered ≥21 Gy to target D98%, but ZAP‐X achieved a higher dose (*p* < 0.05). Target D2% was also higher with ZAP‐X (*p* < 0.05). Cochlear and brainstem doses were comparable (*p* = 0.410 and 0.252, respectively). Skin dose and V7 Gy of normal brain tissue were significantly lower with CK (*p* < 0.05), while V12 Gy was not different (*p* = 0.094). MU was lower with CK, but not significantly (*p* = 0.061). Furthermore, the ID of the normal brain was significantly lower with CK (*p* < 0.05). BT was significantly shorter with CK (*p* < 0.05). Figure 1 illustrates representative cases from the grade I/II group. In both Koos grade I (Patient 2, Target volume 0.22 cc) and grade II (Patient 6, Target volume 0.97 cc), ZAP‐X demonstrated higher CI, lower GI, and reduced cochlear and brainstem doses compared with CK. Figure 2 shows representative grade III/IV group. In Koos grade III (Patient 18, Target volume 2.62 cc) and grade IV (Patient 28, Target volume 8.06 cc), ZAP‐X achieved higher CI, while cochlear dose was similar in grade III and lower with ZAP‐X in grade IV. At low‐dose levels (< 10 Gy), CK exhibited less dose spread.

**TABLE 2:** The mean values and standard deviations (SD) of the dosimetric and delivery parameters for the grade I/ II and grade III/IV groups.

	Grade I/II group			Grade III/IV group		
Parameter	CK Mean ± SD	ZAP‐X Mean ± SD	*p*‐value	CK Mean ± SD	ZAP‐X Mean ± SD	*p*‐value
GI	4.45 ± 0.86	3.16 ± 0.28	<0.001^*^	2.93 ± 0.18	3.23 ± 0.39	0.003^*^
CI	0.62 ± 0.04	0.74 ± 0.04	<0.001^*^	0.74 ± 0.04	0.79 ± 0.03	0.002^*^
Prescription isodose (%)	70 ± 4	57 ± 3	<0.001^*^	69 ± 5	57 ± 3	<0.001^*^
Target *D*_98%_ (Gy)	12.02 ± 0.05	11.83 ± 0.31	0.033^*^	21.06 ± 0.10	21.33 ± 0.21	0.003^*^
*D*_2%_ (Gy)	17.09 ± 1.05	21.10 ± 1.14	<0.001^*^	31.75 ± 2.40	37.17 ± 2.00	<0.001^*^
Cochlea *D*_max_ (Gy)	9.62 ± 2.12	8.46 ± 2.74	0.002^*^	15.56 ± 3.41	14.85 ± 5.20	0.410
Brainstem *D*_1cc_ (cc)	1.96 ± 0.70	1.63 ± 0.61	0.011^*^	11.89 ± 3.03	11.63 ± 2.07	0.252
Skin (3 mm thick) D_10cc_ (cc)	0.44 ± 0.13	0.30 ± 0.13	0.002^*^	1.62 ± 0.49	2.02 ± 0.48	<0.001^*^
Normal brain tissue						
V7 Gy (cc)	1.05 ± 0.89	0.63 ± 0.60	<0.001^*^	24.71 ± 11.12	35.24 ± 17.31	<0.001^*^
V12 Gy (cc)	0.33 ± 0.33	0.26 ± 0.26	0.004^*^	11.86 ± 5.19	11.46 ± 5.24	0.094
Integral dose (Gy·L)	0.27 ± 0.07	0.27 ± 0.10	1.000	1.49 ± 0.42	2.15 ± 0.69	<0.001^*^
Monitor unit	7103.10 ± 1659.58	11332.92 ± 3174.61	<0.001*	17477.83 ± 3637.53	20553.62 ± 3271.63	0.061
Beam‐on time (min)	16.33 ± 5.27	18.60 ± 6.53	0.118	17.93 ± 3.67	23.27 ± 4.84	0.003^*^

* < 0.05.

## 4 DISCUSSION

SRS and SRT have been established as alternative treatment modalities to microsurgery for VS, demonstrating local control rates of over 90%.^25^ SRS has traditionally been performed using GK; however, equivalent results can now be obtained using conventional LINAC and CK.^26^, ^27^ Casentini et al.^28^ reported a 5‐year local control rate of over 80% for SRT using CK, delivered in 2–5 fractions for target volumes of 8–24 cc. Similarly, with CK, Hansasuta et al.^29^ reported local control rates of 99% and 96% at 3 and 5 years, respectively, using 18 Gy/3 fractions. These outcomes are comparable to those of SRS and conventional fractionated radiation therapy. The side effects of SRS include trigeminal and facial nerve dysfunction, dizziness, and gait disturbances.^30^ Furthermore, hearing preservation after treatment is strongly correlated with the radiation dose to the cochlea; however, no significant difference in hearing preservation has been observed between SRS and SRT.^31^, ^32^ In this study, treatment plans for VS using CK and ZAP‐X were compared according to the Koos classification.

Several studies have compared radiation dose distributions among different radiotherapy techniques for VS. Kim et al.^33^ compared SRS plans using VMAT (with 1 coplanar, 3 and 5 non‐coplanar arcs) and GK. VMAT plans delivered a lower target dose (*D*_2%_ and *D*_mean_) and a shorter BT than GK, while CI with VMAT using five non‐coplanar arcs was superior to GK when the target volume exceeded 0.5 cc. Khong et al^5^ evaluated SRS with dynamic conformal arc therapy (DCAT), IMRT, and VMAT, showing that VMAT provided sufficient dose to the target while limiting the dose to the cochlea more than DCAT or IMRT. Lee et al.^17^ compared DCAT and HT, reporting that HT achieved better CI but had a less favorable dose gradient, as well as longer BT and higher MU. Dutta et al.^34^ compared Linac‐based SRS (BrainLAB system plan) with CK and found no significant difference in CI or maximum brainstem dose, but a lower mean cochlear dose with CK. Muacevic et al.^35^ compared CK and ZAP‐X dosimetry in 20 cases and reported significantly higher MU for ZAP‐X. Consistently, in the present study, MU was also higher for ZAP‐X, likely because of its lower beam energy (3 MV), which increases attenuation. Although the previous study found no significant difference in BT, in our cohort CK achieved significantly shorter BT in the grade III/IV group. This may reflect the larger tumor volumes in our study (mean 4.62 cc in grade III/IV vs. a maximum of 1.37 cc in the previous study), which likely required multiple isocenters in ZAP‐X. In both groups, ZAP‐X achieved superior CI, suggesting that ZAP‐X consistently provides better dose conformity regardless of target size. In the previous study, there were no significant differences in GI or OAR doses. In contrast, the grade I/II group showed significantly better OAR sparing and GI values with ZAP‐X, while in the grade III/IV group, GI and skin dose were slightly better with CK (*p* < 0.05), while cochlea and brainstem doses remained comparable. The reason for the GI results of ZAP‐X in this study is thought to be that the maximum energy of 3 MV, which reduces scattered penumbra, and the 450 mm SAD, which results in a smaller geometric penumbra and a steeper target circumference, leading to better GI.^21^, ^36^ These suggest that the quality difference in CK and ZAP‐X treatment plans diminishes as target volume increases. For brain metastases, ZAP‐X has been reported to produce better dose distribution than CK for small target volumes; however, as the target volume increases, this difference becomes smaller, similar to the findings of the present study.^37^ In the present study, the ID to the normal brain did not significantly differ between ZAP‐X and CK in the grade I/II group. In contrast, in the grade III/IV group, the ID to the normal brain was significantly lower with CK than with ZAP‐X. This finding suggests that the effect of beam energy on the ID becomes more prominent as the target volume increases. Previous studies have also reported that higher photon energies reduce the ID to normal tissues. Sung et al.^38^ demonstrated that increasing photon energy from 6 to 10–15 MV reduced the ID to surrounding normal tissues by approximately 7% in IMRT plans. Similarly, Cosset et al.^39^ noted that lower energy photon beams may result in higher stray doses and increased low‐to‐intermediate dose volumes in normal tissues. These findings support the observation that the deeper penetration of higher‐energy beams contributes to improved dose efficiency in larger target volumes, thereby reducing the ID to the normal brain. For small tumors, however, the irradiated volume of normal brain is limited, and the effect of beam energy on the ID may therefore be less pronounced, which explains the absence of a significant difference between 3 and 6 MV in the grade I/II group. However, in comparisons of cases with brain metastases, it has been reported that both MU and BT were more favorable with ZAP‐X than with CK.^40^ These findings suggest that BT and MU outcomes may be influenced by the planning methodology based on target size and shape. For very small tumors, CK can use a 5‐mm collimator, whereas ZAP‐X can use a 4‐mm collimator. Based on the results of this study, ZAP‐X may provide a potential advantage over CK in the treatment of such cases.

This study has several limitations. First, in this study, CK MultiPlan TPS was used for CK planning; however, it is noteworthy that the current CK planning system has transitioned to the Precision TPS (Accuray). This replacement introduces the new VOLO algorithm, which could potentially lead to differences, particularly in BT and MU values.^41^ Second, this study used the CK G4 system, in which only Fixed and Iris collimators are available. Therefore, treatment plans based on the multileaf collimator (MLC) could not be evaluated. A previous study reported that the MLC is dosimetrically feasible for intracranial SRS and can reduce beam delivery time compared with Fixed/Iris plans, although its conformity may be inferior for small targets or when OAR is located adjacent to the target.^42^, ^43^ Thus, our results reflect the current clinical practice with the G4 platform, and future studies including MLC‐equipped CK systems are warranted. Third, although BT was compared, this study did not evaluate the time required for planning, which may impact clinical workflow. Finally, this study did not include pre‐treatment quality assurance (QA). Our analysis focused on comparing treatment planning results, and no measurements of absolute dose or gamma analysis were performed. However, pre‐treatment QA using recommended 3D gamma passing criteria would provide important information regarding the agreement between planned and delivered dose distributions. Future studies incorporating such QA are needed to validate the present findings.

## 5 CONCLUSION

ZAP‐X generally requires longer BT than CK, regardless of target volume. For smaller target volumes, such as Koos grade I/II, ZAP‐X may achieve a superior dose distribution compared with CK. However, for larger targets, as seen in Koos grade III/IV, ZAP‐X can provide treatment plans comparable to CK, although CK is capable of achieving a steeper dose gradient.

## AUTHOR CONTRIBUTIONS

*Study conception and design*: Toshihiro Suzuki, Masahide Saito, Hiroshi Onishi, Ryutaro Nomura, Naoki Sano, Zhe Chen, and Hiroshi Takahashi. *Acquisition of data*: Toshihiro Suzuki, Masahide Saito, Ryutaro Nomura, Hikaru Nemoto, and Ryuma Sawada. *Analysis and interpretation of data*: Toshihiro Suzuki, Masahide Saito, Ryutaro Nomura, Hikaru Nemoto, and Ryuma Sawada. *Drafting of manuscript*: Toshihiro Suzuki, Masahide Saito, Hiroshi Onishi, Masahide Saito, Ryutaro Nomura, Hikaru Nemoto, Ryuma Sawada, Zennosuke Mochizuki, Tran Van Ton, and Hiroshi Takahashi. *Critical revision*: Toshihiro Suzuki, Masahide Saito, Hiroshi Onishi, and Hiroshi Takahashi.

## CONFLICT OF INTEREST STATEMENT

There are no conflicts of interest to declare.

## ETHICS STATEMENT

This study was approved by the institutional review board (IRB) of Kasugai General Rehabilitation Hospital (receipt number: 2024‐2)

## Data Availability

All data generated and analyzed during this study are included in this published article (and its supporting information files).
